# Editorial: The interplay of gut-microbiome between infection and inflammation

**DOI:** 10.3389/fcimb.2024.1413473

**Published:** 2024-06-06

**Authors:** Piyush Baindara, Santi M. Mandal

**Affiliations:** ^1^ Animal Sciences Research Center, Division of Animal Sciences, University of Missouri, Columbia, MO, United States; ^2^ Department of Biotechnology, Indian Institute of Technology Kharagpur, Kharagpur, WB, India

**Keywords:** gut-microbiome, infection, inflammation, autoimmune diseases, cancer

Because of its varied makeup and role in regulating human health and disease, the gut microbiome has drawn huge attention from the scientific community in recent years. As a result of scientific advancements including high-throughput genome sequencing and transcriptome analysis, the gut microbiome has become a viable therapeutic target for both infectious and autoimmune diseases. In particular, it has been demonstrated that respiratory infections, such as COVID-19 and tuberculosis, significantly influence the pathogenesis of the disease progression due to gut dysbiosis, which in turn impacts the regulation of the gut-lung axis and advances the disease (Baindara et al., 2021). Conversely, it has been noted that gut dysbiosis plays a significant role in several chronic conditions, such as cancer, diabetes, obesity, inflammatory bowel disease, neurological disorders, cardiovascular complications, biliary diseases, colitis, appendicitis, and metabolic disorders (De Vos et al., 2022; Hou et al., 2022). Despite the acknowledged involvement of the gut microbiome in various pathologies, it is currently unclear how the gut microbiota contributes to the development and regulation of various diseases. Infection-associated inflammation or sterile inflammation and its molecular interaction with gut microbiota and gut dysbiosis, along with overall disease progression is one of the exciting research areas that requires further detailed investigation to understand the unanswered questions. Furthermore, understanding and methods to overcome gut dysbiosis, gut microbiome transfer, gut microbiota-based host-directed therapies, understanding the impact of conventional antibiotics on the gut microbiome, and drug-repurposing techniques to alter the gut microbiome are key underexplored Research Topics on the path to scientific advancement in the role and regulation of gut microbiome research.

Acting as a bridge between the liver and the intestine, the biliary system demonstrates proximity to the gut microbiome. Importantly, the biliary system plays an essential role in metabolic regulation, especially in lipid metabolism in the bile duct. The occurrence of biliary system diseases has rapidly increased in recent years, but the involvement of the microbiota has not yet been fully explored in this regard. In a recent systemic review, Wang et al. reviewed the associated microbial diversity in patients with gallstones (GS), primary sclerosing cholangitis (PSC), primary biliary cholangitis (PBC), and biliary tract cancer (BTC). Systemic analysis revealed that the prevalence of the genus *Faecalibacterium* was decreased in GS, PSC, PBC, and BTC patients, whereas the abundance of the genera *Veillonella*, *Lactobacillus*, *Streptococcus*, and *Enterococcus* was noticeably increased in the cases of PSC, PBC, and BTC-associated patients. Remarkably, the diversity of the genus *Clostridium* was found to be decreased in GS, PBC, and BTC patients while it was generally increased in PSC patients. Another study by Chen et al. explored the microbiome associated with biliary obstruction in patients infected with *Clonorchis sinensis*. An increase in *Proteobacteria* and a decrease in *Firmicutes* were reported in the biliary microbiome of *C. sinensis*-infected patients at the phylum level. Also, a significant increase in the genera *Pseudomonas* and *Staphylococcus* was observed along with a noticeable reduction in *Enterococcus* bacteria at the genus level in infected patients. Furthermore, lying within proximity, the gut microbiome was found to have a significant role in the liver environment via the gut-liver axis. Any changes in the gut microbiota can induce liver inflammation, which can further result in chronic liver diseases (CLDs) such as liver cirrhosis (**Figure 1**). Next, Wu et al. studied the distribution of the gut microbiome in patients with liver cirrhosis by employing 16S rRNA gene sequencing of stool samples. The results demonstrated a prominently reduced microbial diversity and diminished abundance of typical SCFA-producing bacterial groups including *Firmicutes*, *Coprococcus*, and *Clostridium* IV, along with an enhanced burden of pathogenic bacterial groups, *Gammaproteobacteria*, *Veillonella*, and *Bacilli* in cirrhotic patients. It has been suggested that probiotics may be used as an adjuvant therapy to boost SCFA-producing bacteria in patients with liver cirrhosis.

A gut infection caused by *Clostridioides difficile* is a major cause of nosocomial diarrhea. A microbial profiling study by Vázquez-Cuesta et al. reported an increased burden of *Fusobacterium*, and a reduction of *Collinsella*, *Senegalimassilia*, *Prevotella*, and *Ruminococcus* in recurrent *C. difficile* infection (R-CDI). Interestingly, the rate of R-CDI was significantly correlated with increased levels of calprotectin (**Figure 1**). The authors suggested a predictive model for R-CDI, employing dominant microbial genera and calprotectin levels.

In addition to infection-associated gut dysbiosis and involvement in the inflammatory response, the gut microbiome has also been reported to play an important role in the progression of cancers such as colorectal cancer (CRC). A study by Bucher-Johannessen et al. showed an abundance of *Phascolarctobacterium succinatutens*, *Bifidobacterium*, and *Lachnospiraceae* spp. associated with the patient samples diagnosed with CRC and high-risk adenoma (HRA). In another study, Xu et al. evaluated the dynamic changes of migratory microbial components in colonic tissue using an LPS-induced rat model of sepsis. The results of this study determined the temporal dynamics of bacteria and bacterial translocation from various sources to colonic tissues at different time points of sepsis progression (**Figure 1**). In conclusion, the identified bacterial migrants may serve as potential biomarkers that directly or indirectly alter the structure and function of the colonic microbiota, during the pathophysiology of sepsis.

It is a known fact that during COVID-19, gut microbiota played an important role in disease regulation by modulating the gut-lung axis. However, children showed only mild symptoms. To uncover the protective effect of the gut microbiome in children during COVID-19, Marzano et al. performed a metaproteome analysis of the gut microbiota in pediatric patients. Univariate analysis revealed that the pediatric gut microbiota played a protective role by being involved in metabolic processes such as tryptophan, butanoate, fatty acids, and bile acid biosynthesis along with antibiotic resistance and virulence. Another, meta-analysis by Reuben et al. analyzed the oral and nasopharyngeal or gut microbiome in COVID-19 patients and found a prominent reduction in the gut microbial diversity but not in the respiratory microbiome. Furthermore, Petakh et al. reported on the role of gut microbiota in COVID-19 progression in Type 2 diabetes (T2D) patients (**Figure 1**). The results showed a positive correlation between specific gut microbiota (*Klebsiella* spp. and *Enterococcus* spp.) and C-reactive protein levels and length of stay in COVID-19 patients with or without T2D. Overall, this study revealed the potential role of specific gut microbiota in COVID-19 patients with T2D, which can be used to develop treatment strategies for COVID-19 and associated T2D.

In the recent past, the impact of the gut microbiota on cardiovascular disease has also been demonstrated. An interesting review by Wang et al. explored the gut-heart axis and its role in myocarditis along with detailing the cross-linking of the immune system with the gut microbiota, and its metabolites. Next, Zhang et al. studied the gut microbiome in infants with congenital heart disease and showed the association between heart failure and the gut microbiome. The results suggested an abundance of *Firmicutes*, *Actinobacteria*, *Proteobacteria*, and *Bacteroidetes* at the phylum level and *Enterococcus*, *Bifidobacterium*, *Subdoligranulum*, *Shigella*, and *Streptococcus* at the genus level in the heart failure group of infants (**Figure 1**). Moreover, a noticeable reduction was observed in the alpha and beta diversity of the gut microbiome in the heart failure group in comparison to the control group along with a downregulation of the retinol metabolism. Overall, it is suggested that the gut microbiome could serve as a potential biomarker for the early diagnosis of heart disease.

The azomethane-dextran sodium sulfate (AOM-DSS) mouse model is well-established for the induction of acute colitis, but the success rate has always been an issue. A study of the gut microbiome associated with the AOM-DSS mouse model by Sun et al. revealed the important role of gut microbiota at the early stage of model development to improve the success rate of model construction. Gut microbiome analysis revealed uncontrolled proliferation of *Pseudescherichia*, *Turicibacter*, and *Clostridium* XVIII leading to death. A significant reduction was observed in the genera *Ligilactobacillus*, *Lactobacillus*, and *Limosilactobacillus* while *Akkermansia* and *Ruthenibacterium* were found to be increased in the gut of live AOM-DSS mice. The involvement of *F. nucleatum* in the progression of colitis has also been reported in the recent past. Taking this further, Duan et al. explored the role of fucose in the mice treated with *F. nucleatum* or fucose-treated *F. nucleatum* followed by colitis induction using DSS. The results suggested a protective role for fucose in colitis as the group of mice injected with fucose-treated *F. nucleatum* showed a reduced inflammatory response. It is revealed that the metabolic pathways of *F. nucleatum* are altered upon fucose treatment resulting in reduced pro-inflammatory properties of *F. nucleatum*. Overall it is suggested that fucose could be used as a functional food or prebiotic for the treatment of *F. nucleatum-*induced colitis. Next, a two-sample Mendelian Randomization (MR) study by Zhang et al. explored the relationship between gastroduodenal ulcers and gut microbiota employing a genome-wide association study (GWAS). The results demonstrated a negative correlation of gastroduodenal ulcers with the prevalence of *Enterobacteriaceae*, *Butyricicoccus*, *Candidatus soleaferrea*, *Lachnospiraceae* NC2004, *Peptococcus*, and *Enterobacteriales* while a positive correlation was observed with the abundance of *Streptococcaceae*, *Lachnospiraceae* UCG010, *Marvinbryantia*, *Roseburia*, *Streptococcus*, *Mollicutes* RF9, and NB1n. Overall, this study provides an important perspective for the gut microbiota-based therapeutic approaches for gastroduodenal ulcers. Similarly, another two-sample MR study by Wang et al. investigated the relationship between appendicitis and gut microbiota using GWAS. Their analysis revealed an inverse association of appendicitis risk with the prevalence of the genera *Deltaproteobacteria*, *Christensenellaceae*, *Desulfovibrionaceae*, *Eubacteriumruminantium*, *Lachnospiraceae* NK4A136, *Methanobrevibacter*, *Desulfovibrionales*, and *Euryarchaeota* while a positive correlation was found with the increased number of Family XIII, *Howardella*, and *Veillonella* which are associated with appendicitis susceptibility (**Figure 1**).

Interestingly, the gut microbiota has also been reported to be involved in dysbiosis-associated depression, but the detailed mechanism is unknown. An exciting study by Huang et al. examined chronic unpredictable mild stress (CUMS) and its association with the gut microbiome and NLRP3 inflammasome using fecal transplantation (FMT). It was found that FMT from CUMS rats to the antibiotic-treated rats showed enhanced NLRP3 inflammasomes along with increased levels of inflammatory cytokines. Moreover, probiotic treatment successfully balanced the altered gut microbiome induced by CUMS treatment and resulted in a diminished inflammatory response. Gut dysbiosis is also known to be associated with autoimmune diseases and associated inflammation (De Luca and Shoenfeld, 2019; Baindara, 2024). Chang and Choi recently reviewed the correlation of the gut microbiome with autoimmune diseases, especially focusing on Sjögren’s syndrome (SS), systemic lupus erythematosus (SLE), rheumatoid arthritis (RA), and multiple sclerosis (MS). The study suggests that gut dysbiosis in autoimmune diseases plays an essential role in the immune response by maintaining gut homeostasis, and so associated gut microbiota could be used as biomarkers for the treatment and early diagnosis of autoimmune diseases (**Figure 1**).

Overall, these studies have demonstrated the interplay of gut microbiota and defined microbial biomarkers in multiple infectious and autoimmune diseases (**Figure 1**). It is strongly recommended that specific gut microbiota could be used as therapeutic biomarkers in the early diagnosis of associated diseases. In addition, a general enrichment of the gut microbiome using probiotics could help reduce the incidence of diseases.

**Figure 1:**
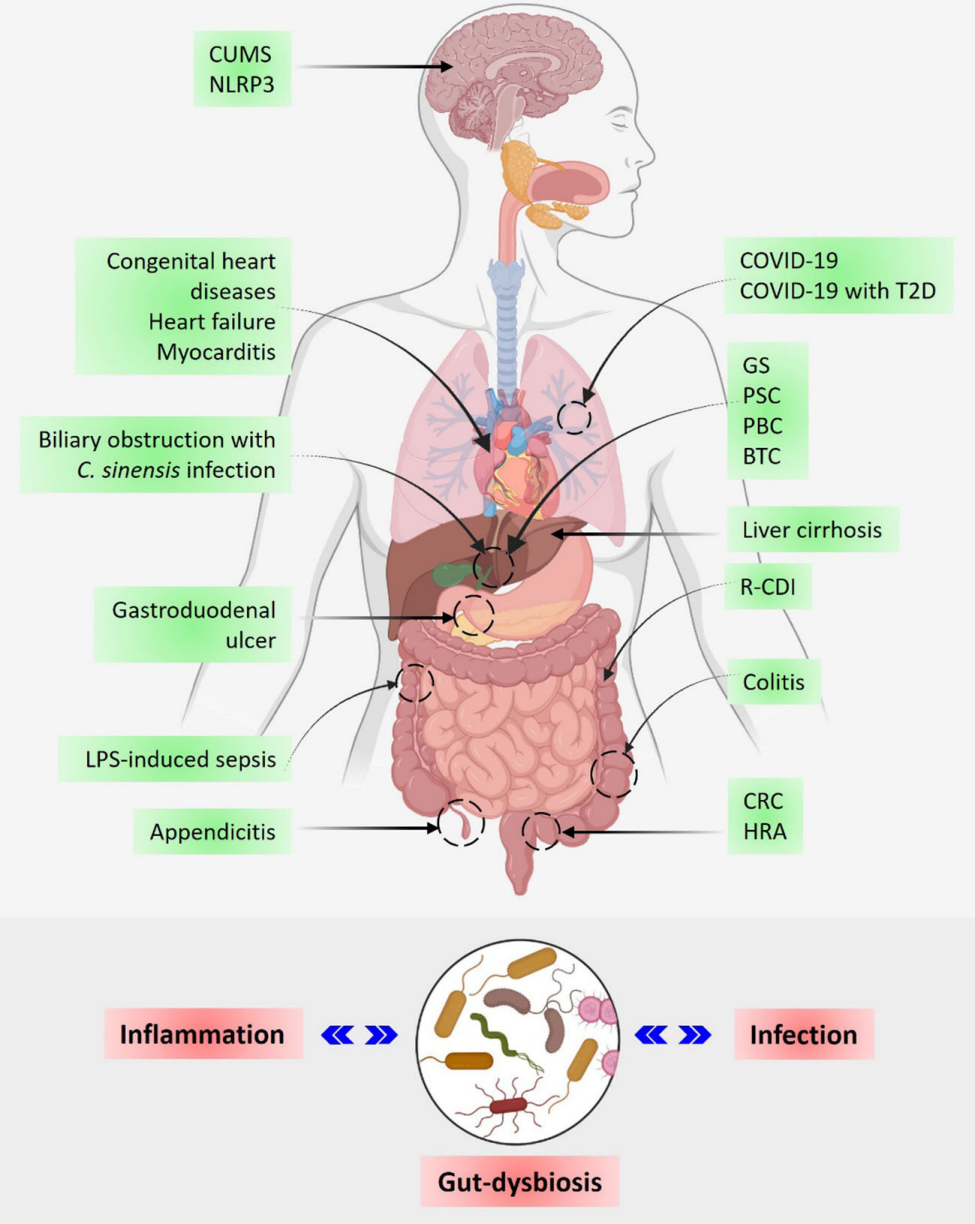
The gut microbiome in human health and disease. The upper panel shows the involvement of gut microbiota in the regulation of infectious and autoimmune diseases including cancer. The lower panel shows a schematic representation of the inverse relationship between inflammation, gut dysbiosis, and infection. CUMS, Chronic unpredictable mild stress; T2D, Type 2 diabetes; GS, Gallstone; PSC, Primary sclerosing cholangitis; PBC, Primary biliary cholangitis; BTC, Biliary tract cancer; R-CDI, *C. difficile* infection; CRC, Colorectal cancer; and HRA, High-risk adenoma.

## Author contributions

PB: Conceptualization, Investigation, Project administration, Supervision, Validation, Visualization, Writing – original draft, Writing – review & editing. SM: Investigation, Supervision, Validation, Writing – review & editing.
